# It’s not what it looks like: challenges in diagnosis of synovial lesions of the knee joint

**DOI:** 10.1186/s40634-016-0039-3

**Published:** 2016-01-29

**Authors:** Kumar Kaushik Dash, Piyush Vishwas Gavai, Roshan Wade, Amyn Rajani

**Affiliations:** Department of Orthopaedics, Grant Government Medical College & Sir J.J. Group of Hospitals, Byculla, Mumbai, India; Department of Orthopaedics, Seth G.S. Medical College & K.E.M. Hospital, Parel, Mumbai, India; Breachcandy Hospital & Saifee Hospital, Mumbai, India

## Abstract

**Background:**

With the advent of MRI (Magnetic Resonance Imaging), Synovial lesions around knee are being more and more easily detected. Synovial lesions of knee present with boggy swelling, effusion, pain, and restriction of motion. Differential diagnoses of such lesions include pigmented villonodular synovitis, synovial lipoma, synovial chondromatosis, rheumatoid arthritis, synovial hemangioma, amyloid arthropathy, xanthomata and lipoma arborescens. CT and MRI often help in diagnosis of such lesions. MRI of Lipoma Arborescens has been regarded to have characteristic diagnostic appearance – it includes a synovial mass with frond-like architecture and fat signal intensity on all pulse sequences. Sometimes Lipoma Arborescens can present in conjunction with inflammatory arthritis. Synovectomy is often curative for such conditions.

**Findings:**

We report two cases where lesions diagnosed as Lipoma Arborescens on MRI subsequently revealed to be chronic inflammatory synovitis, characterized by absence of fat infiltration in histopathological examination – refuting the original diagnosis. There was infiltration of lymphocytes and neutrophils in the synovium, suggestive of chronic inflammatory arthritis. Both of these patients required management from rheumatologist, and had relief of symptoms after use of methotrexate and hydroxychloroquine. We also report a third case, where a loose body appearing as chondral fragment on arthroscopy was subsequently diagnosed as an organized hematoma on histopathological examination.

**Conclusion:**

Diagnostic pitfalls after MRI of the knee is not uncommon. For example - normal variant of meniscomeniscal ligaments have been reported as meniscal tears; motion artifacts have been falsely reported as meniscal injuries; and meniscofemoral ligament can appear as free osteochondral fragment. In most of these cases, a routine arthroscopy is enough to clear the confusion. However, as evident in the three cases described here - in some synovial lesions of knee joint, even after MRI and arthroscopic examination, histopathological confirmation may still be prudent. In spite of availability of advanced imaging technologies and high definition arthroscopy equipment, an arthroscopy surgeon still must not forget the value of histopathological examination in establishing the true nature of synovial lesions of the knee joint.

## Introduction

Tools and technologies available for diagnosis and management of orthopaedic conditions have come a long way in last few decades. With advent of MRI (Magnetic Resonance Imaging), soft tissue lesions are being more and more easily detected. Magnetic Resonance Imaging (MRI) is often precedes arthroscopic procedures on the knee, and many times, management decisions are based upon MRI. Modern MRI uses superconducting magnets and radiofrequency coils. This produces a high contras image of the bone and soft tissues via manipulation of hydrogen protons (Hartley et al. 2012).

Histopathology, on the other hand, has been playing significant role in clinical medicine since as early as 19^th^ century (Musumeci 2014). And after all these years, it still remains the tool for ultimate diagnosis of bone and soft tissue growths and tumors (Traina et al. 2015).

While utilizing the armamentarium of various diagnostic tools available to the orthopaedic surgeon, we must be careful to always remember the fundamentals. There are certain situations, where we already know that MRI can be less accurate; for example, soft tissue sarcomas (Patel et al. 2015), meniscal tears (Huber & Trieb 2002) and osteonecrosis (Trepman & King 1992). We can call these as known unknowns. We as surgeons ‘know’ to take additional precautions in these scenario. However, there exists a subset of unknown unknowns; i.e., pathologies where the surgeon is not aware that MRI can lead to misdiagnosis. For example, for certain conditions (such as Lipoma Arborescens), the characteristic MRI appearance has been described as diagnostic (Kloen et al. 1998). If it were to happen where the MRI imaging would misdiagnose any of those pathologies, then the surgeon would be left with a difficult situation if a confirmation histopathology has not been done.

We describe a case series comprising of two cases diagnosed as Lipoma Arborescens and one case diagnosed as Nodular Synovitis; where histopathology refuted the original diagnosis and changed the plan of management.

## Findings

**Fig. 1:**
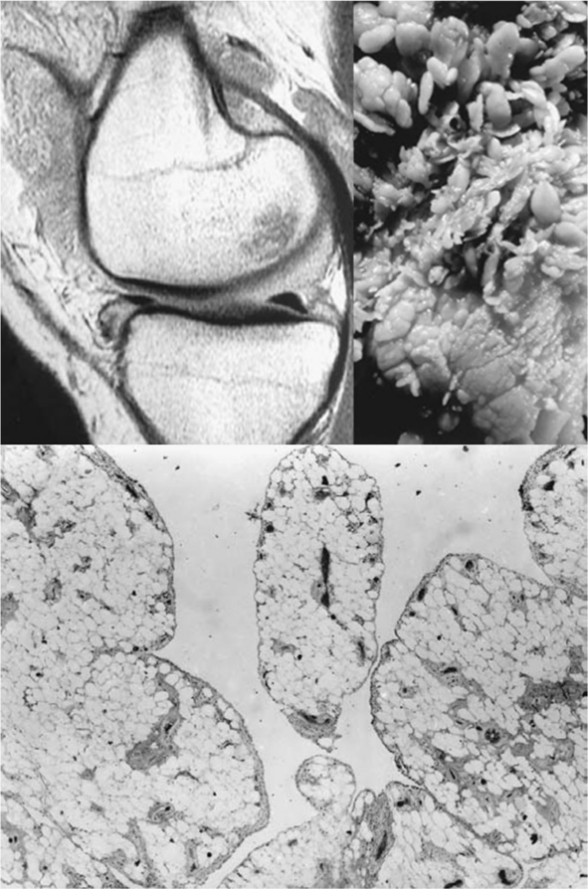
MRI Picture, Gross Appearance & Histopathological Impression of Lipoma Arborescens: clockwise from top left (villous like projection in the synovium of suprapatellar pouch with signal density characteristic of fat; photograph showing villous architecture of synovium, histomicrograph demonstrating adipose tissue in the subsynovium leading to villous expansion of the synovium. [Source: Kloen et al, Lipoma Arborescens of the Knee. Journal of Bone and Joint Surgery (Br) 1998:80-B, 298-301] (Kloen et al. 1998)

Lipoma Arborescens of the knee joint usually presents as long-standing, gradually progressive swelling, and is often associated with joint effusion, pain and restriction of range of motion. Kloen et al have described 6 such cases, with characteristic MRI picture, arthroscopic appearance and histopathological confirmation (Kloen et al. 1998) (Fig. 1). All cases were treated with synovectomy.

### Case 1

A 28-Year-old male presented with dull aching pain and swelling in right knee since 2 and a half years. The symptoms were not relieved by intermittent intakes of oral analgesics over this time period. On clinical examination, effusion of knee joint was noted, along with boggy consistency on palpation suggestive of synovial hypertrophy. There was no joint line tenderness. McMurray test failed to elicit any pain. Tests for instability (Drawers, Lachman’s & Varus-Valgus stress) were negative. Plain x-ray image was unremarkable apart from enlarged soft tissue shadows.

**Fig. 2:**
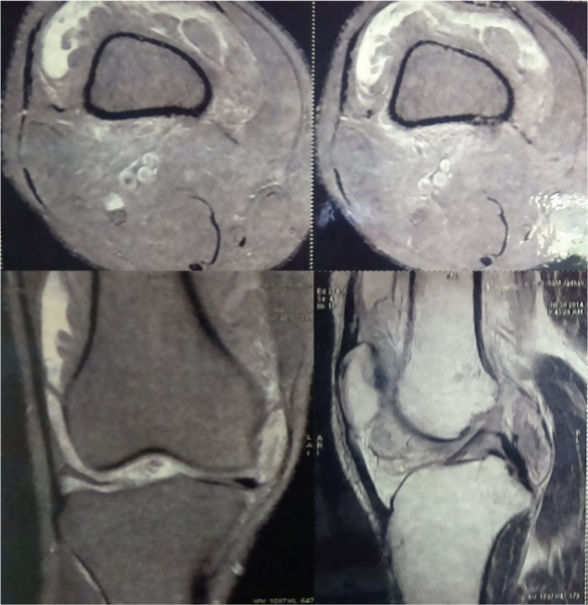
MRI Image of Knee showing Frond-like synovial proliferative growth (Case 1) clockwise from top left (transverse image showing synovial proliferation around distal femur; transverse image showing synovial effusion in suprapatellar pouch; coronal image showing frond like proliferation both medially and laterally; sagittal image showing involvement of patellofemoral space and posterior capsular region

MRI report concluded Lipoma Arborescens of the synovium of knee joint with characteristic synovial growth pattern; and associated moderate synovial joint effusion extending in to suprapatellar bursa. There were focal benign lesions/marrow edema in the femoral intercondylar region and medial femoral condyle (Fig. 2).

**Fig. 3:**
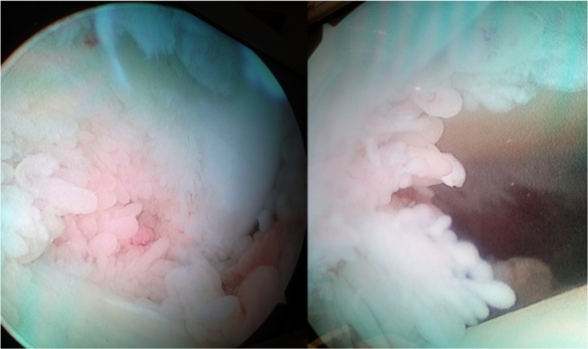
Arthroscopic image of synovial growth, showing frond like synovial proliferation extending in to tibiofemoral space (Case 1)

As patient was not responding to analgesics and conservative management, Arthroscopic Synovectomy was undertaken. Intra-operative images showed synovial hypertrophy with branching frond like pattern (Fig. 3).

**Fig. 4:**
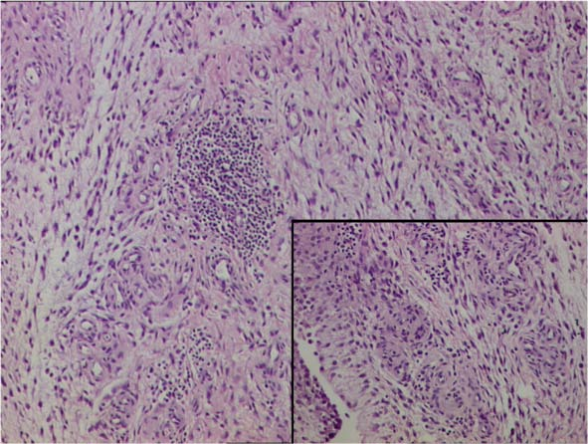
Histopathology reports showing fibrocollagenous tissue with dense mixed inflammatory infiltrate comprised of lymphocytes, plasma cells, neutrophils and proliferative blood vessels. There was no fat infiltration in the synovial tissue

**Fig. 5:**
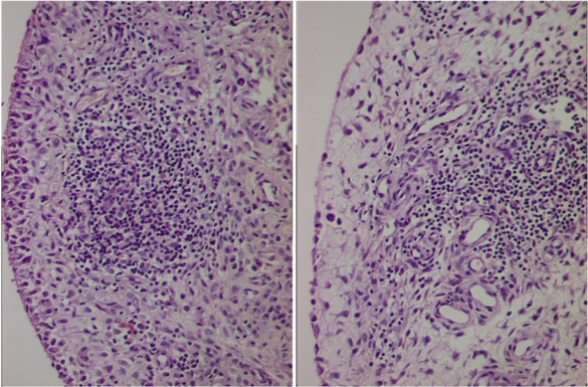
Histopathology reports showing lack of any fat infiltration in the synovial tissue

Extensive synovectomy was done, clearing all compartments of knee from the growth. Samples of the tissue were sent to two different labs for histopathology. Histopathology reports showed fibrocollagenous tissue with dense mixed inflammatory infiltrate comprised of lymphocytes, plasma cells, neutrophils and proliferative blood vessels. For comparison, histopathology of Lipoma Arborescens normally shows synovial villiform structures with mature fat cell proliferation in the sub-synovial layer (Kloen et al. 1998; Kamaci et al. 2015). There was no fat infiltration in the synovial tissue in our case, thus excluding possibility of Lipoma Arborescens (Figs. 4 and 5). The pathologist concluded it as suggestive of chronic inflammatory synovitis, with possibility of rheumatoid arthritis.

The patient was referred to Rheumatology clinic where he was diagnosed with Mono-articular Chronic Inflammatory Synovitis and started on a regimen of methotrexate (7.5 mg once a week), sulfasalazine (1gm twice a day) and indomethacin (25 mg thrice a day). At 1-year follow-up, the patient is completely pain free and able to daily activities. He is still on anti-inflammatory medications.

### Case 2

**Fig. 6:**
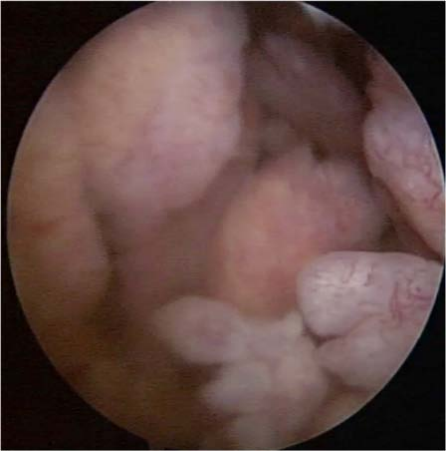
Arthroscopic image of synovial growth, showing villi like proliferation (Case 2)

**Fig. 7:**
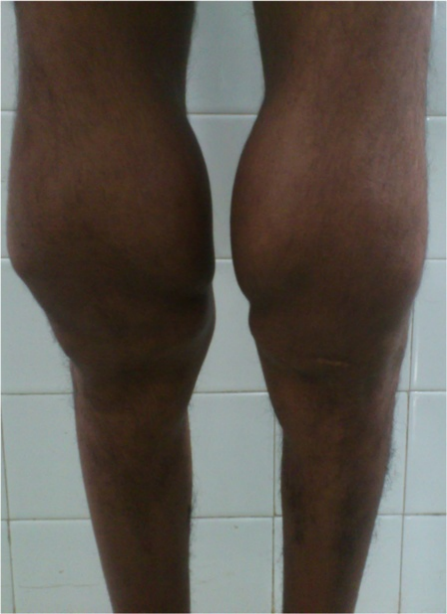
Clinical photograph of bilateral knee joint swelling

A 22-year-old male presented to us with complaint of dull aching pain in the right knee. On Clinical examination, there was effusion, associated with boggy swelling on palpation. MRI report concluded Lipoma Arborescens of the knee. Arthroscopy showed frond-like branching villous synovial growth (Fig. 6). Histopathology indicated chronic inflammatory synovitis. The joint fluid tested positive for Rheumatoid Arthritis IgM (1:40) by Latex Agglutination method. Patient soon developed pain and swelling in opposite knee and both wrists (Fig. 7). Patient was referred to Rheumatology clinic where he was started on methotrexate and hydroxychloroquine. At 4-year-follow-up, patient is pain free at present, with no functional limitation. He is still on rheumatologic medications and undergoes reassessment every 6 months at the rheumatology clinic.

### Case 3

**Fig. 8:**
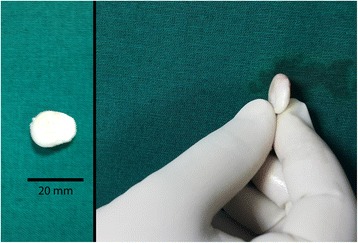
Loose body retrieved arthroscopically (Case 3)

**Fig. 9:**
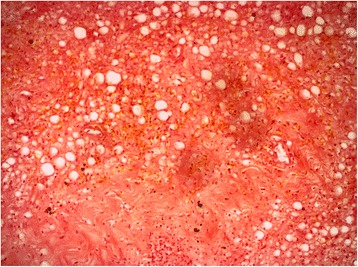
Histopathology revealing hyalinised fibrous nodular mass, with underlying stroma showing an organized hematoma (Case 3)

A 37 years old male patient came with feeling of some moving object in knee, with occasional episodic restriction in movement. It was associated with pain and swelling. X-ray showed normal alignment and joint space. MRI confirmed minimal joint effusion, with a rounded hypointese body in suprapatellar bursa (17 x 16 x 7 mm) suggestive of a focus of nodular synovitis. Arthroscopic surgery was undertaken to remove the body. On macroscopic examination, the loose body hade a white and glistening appearance, like a cartilaginous fragment (Fig. 8). However, even after extensive diagnostic rounds, no cartilage defect could be discovered. The sample was sent for histopathology, which revealed hyalinised fibrous nodular mass, with underlying stroma showing an organized hematoma (Fig. 9). The patient was relieved of his symptoms and is at full functional capacity now, at 1-month follow-up.

A summary of all three cases in compiled in Table 1.

**Table 1:** Summary of all three cases

Case	Age & gender	Symptoms	Physical findings	MRI findings	Treatment and arthroscopy findings	Original diagnosis	Definitive histopathological findings	Final result & follow-up
1	28/Male	Swelling since 2 and a half years with dull aching pain	Knee effusion with boggy swelling. No joint line tenderness. No Instability,	MRI - Frond like synovial growth pattern characteristic of Lipoma Arborescens. Focal marrow edema in intercondylar notch and medial femoral condyle. Moderate synovial effusion	Synovial hypertrophy with branching frond-like pattern noted. Synovectomy done.	Lipoma Arborescens	Fibrocollagenous tissue with dense mixed inflammatory infiltrate comprised of lymphocytes, plasma cells, neutrophils and proliferative blood vessels.	Patient is being treated for Chronic Inflammatory Synovitis. Patient continues to have improvement in symptoms while being on anti-inflammatory medications.
2	22/Male	Dull aching pain and swelling.	Effusion and boggy swelling.	Frond like branching synovial proliferation. Knee effusion.	Frond-like branching synovial growth. Synovectomy done.	Lipoma Arborescens	Inflammatory infiltrate in synovium, with absence of adipose tissue proliferation in subsynovial layer.	Patient is being treated for Chronic inflammatory synovitis. Patient is on rheumatologic medications and has improvement of symptoms.
3	37/Male	Pain, Swelling, occasional locking of knee.	Minimal effusion. No tenderness. No instability.	Minimal joint effusion, with a rounded hypointese body in suprapatellar bursa (17 x 16 x 7 mm) suggestive of a focus of nodular synovitis.	White, glistening loose body appearing like a cartilaginous tissue. Loose body removal done.	Unclear Diagnosis. Differentials included Nodular Synovitis or Cartilage injury.	Hyalinised fibrous nodular mass, with underlying stroma showing an organized hematoma	Patient has no complaints at present after removal of the loose body.

## Discussion and review of literature

Diagnostic pitfalls after MRI of knee is not uncommon. However, the literature is minimal on the situations where there was need for histopathology after knee arthroscopy, resulting in change of diagnosis and management. Sanders et al have reported normal variant oblique meniscomeniscal ligament being falsely reported as displaced flap tear of posterior horn of lateral meniscus (Sanders et al. 1999). Mirowitz has reported about motion artifact during MRI being misinterpreted as meniscal tears (Mirowitz 1994). The same danger is also possible due to truncation artifacts (Turner et al. 1991). Meniscofemoral ligament has been reported to falsely provide an MRI appearance of free osteochondral fragment or meniscal tear (Carpenter 1990). Anterior horn of lateral meniscus is known to give high signal near its central attachment, falsely suggesting a tear (Shankman et al. 1997). The limitations of MRI in such scenarios is at least well known, if not well understood by orthopaedics surgeons. And in most of these situations, a routine diagnostic arthroscopy is able to refute the original diagnosis. We, on the other hand, want to highlight on the situations where MRI has been described to be either diagnostic or to have good accuracy, and arthroscopy is not significantly contradictory to MRI findings – but histopathology eventually saves the day by providing the real diagnosis.

The appearance of Lipoma Arborescens on MRI includes a synovial mass with frond-like architecture and it has been considered diagnostic (Kloen et al. 1998). Most of the published literature agrees that the characteristic MRI appearance is pathognomonic and synovectomy is most often curative (Liddle et al. 2012). We show in this case series that not all cases, which appear as Lipoma Arborescens on MRI, have fat infiltration in synovial tissue - thus negating the radiological diagnosis. In both the cases, the histopathology changed our plan of management. Synovectomy alone is sufficient for management of Lipoma Arborescens, but in case of inflammatory arthritis, the patient usually requires prolonged therapy and monitoring from a rheumatologic service.

In addition to the separate entities of idiopathic lipoma arborescens and inflammatory synovitis, newer literatures have started reporting about a new entity – lipoma arborescens coexisting or arising in cases of inflammatory arthritis. Yacyshyn et al have reported a case of recurrent knee effusion with positive cyclic citrullinated peptide in conjunction with lipoma abrorescens (Yacyshyn & Lambert 2010). Coll et al have reported a case with bilateral knee pain swelling associated with symmetric polyarthritis of hands, wrists etc (Coll et al. 2011). Patient underwent open synovectomy and histopathology confirmed elongated synovial folds distended by mature fat cells – diagnostic for lipoma arborescens. Howe et al (Howe & Wenger 2013) have described a series of 45 cases and suggested typical and atypical disease presentations of lipoma arborescens – depending on associated chronic joint inflammation. We could not find any other citation mentioning variation in diagnosis of Lipoma Arborescens through MRI. Although Howe et al (Howe & Wenger 2013) have described atypical forms of LA affecting multifocal presentation, all cases were diagnosed based on MRI examination. A known history of inflammatory arthritis was present in some patients of both ‘typical’ and ‘atypical’ group. The difference between those was not statistically significant. However, there is no mention of whether histopathology was done to confirm the diagnosis after MRI in that study.

This polyarticular clinical presentation described by Coll et al is similar to our Case 2. Furthermore, synovial fluid analysis is positive for RA Factor in our case. However, the negative histopathology report in our case refutes the diagnosis. For a case to be labeled as Lipoma arborescens, it must have fat infiltration in the synovial tissue. In both our cases, this feature was absent. Inflammatory infiltrate with lymphocytes, plasma cells, neutrophils and proliferating blood vessels confirmed chronic inflammatory synovitis. Hence it is possible for inflammatory synovitis to ‘mimic’ the appearance of lipoma arborescens on MRI, with histopathology coming in to clinch the diagnosis. In case 3, we want to highlight the fact that an organized hematoma can appear shiny glistening white, like a chondral loose body, during arthroscopic procedure (Fig. 7). However, the histopathology report allayed our fears of cartilage defect or chondral fracture, revealing the tissue to be an organized hematoma.

Apart from the small sample size and retrospective nature, our study has one additional limitation. It can not be completely ruled out that there could be a problem with the MRI read-out by the radiologist. We have three points to discuss on this issue. First, the two cases of Lipoma Arborescens were reported at two different MRI centers. So it is less likely that the same problem with read-out happened at both the centres. Second, even if we assume that the problem lies in the read-out and not the MRI per se, then also in the end. the orthopaedic surgeon faces the same problem. If the diagnostic test is ambiguous enough to create scenarios of mis-read in any particular pathology, then it is wise for a surgeon to have a secondary safety-net in the form of histopathology. Third, we believe sending the MRI Scans for re-reporting or second opinion will itself lead to an observer bias in this case, and is not practical in routine day-to-day practice. Perhaps a better solution is to conduct a blinded and structured study, to look for inter-observer and intra-observer variability of MRI imaging modality.

## Conclusions

Before the days of MRI, diagnosis of soft tissue lesions like Lipoma arborescens used to be very difficult. Histopathology was needed for confirmation of the diagnosis. But with the advent of newer imaging techniques, battery of blood investigations and high-definition arthroscopy equipment, it is not uncommon for role of histopathology to be sidelined in some pathologies. However, it is prudent for an arthroscopy surgeon to always keep this tool handy, and use it whenever in doubt. Ultimately, knee arthroscopy is a useful tool inspect the joint and to retrieve tissue to be sent for histopathological examination, either to prove the diagnosis of lipoma arborescens or to establish the true nature of the synovial lesion.

### Consent

Written informed consent was obtained from the patient for the publication of this report and any accompanying image.
